# Is the type of school uniform linked with physical activity levels and physical fitness in schoolchildren? A systematic review covering 1,098,972 children and adolescents

**DOI:** 10.3389/fspor.2025.1569511

**Published:** 2025-12-05

**Authors:** Jorge Olivares-Arancibia, Arturo Prat-Lopicich, Rodrigo Yáñez-Sepúlveda, Joaquín González, Boryi A. Becerra-Patiño, Júlio Brugnara Mello, Javier Sanchez-Martinez, Paulo Henrique Guerra, José Francisco López-Gil, Anelise Reis Gaya

**Affiliations:** 1 AFySE Group, Faculty of Education, Research in Physical Activity and School Health, School of Physical Education, Universidad de las Américas, Santiago, Chile; 2 Physical Education, Universidad Metropolitana de las Ciencias de la Educación, Ñuñoa, Chile; 3 Faculty Education and Social Sciences, Universidad Andres Bello, Viña del Mar, Chile; 4 Faculty of Sciences, School of Kinesiology, Universidad de La Serena, La Serena, Chile; 5 Faculty of Physical Education, Universidad Pedagógica Nacional, Bogotá, Colombia; 6 EFiDac Research Group, Pontificia Universidad Católica de Valparaíso, Valparaíso, Chile; 7 Department of Physical Education and Sports, Faculty of Sport Sciences, Sport and Health University Research Institute (iMUDS), University of Granada, Granada, Spain; 8 Instituto de Biociências, Universidade Estadual Paulista, Rio Claro, São Paulo, Brasil; 9 School of Medicine, Universidad Espíritu Santo, Samborondón, Ecuador; 10 Vicerrectoría de Investigación y Postgrado, Universidad de Los Lagos, Osorno, Chile; 11 Projeto Esporte Brasil (PROESP-Br), Universidade Federal de Pelotas, Pelotas, Brazil

**Keywords:** school uniform, sport uniforms, physical activity, schoolchildren, physical fitness

## Abstract

**Introduction:**

School uniforms play a crucial role in promoting physical activity among children and adolescents during school hours. This increased activity can help reduce the risk of metabolic and non-communicable diseases, with physical inactivity and a sedentary lifestyle acting as mediators in the development of these conditions. This systematic review aimed to explore the differences in physical activity levels and physical fitness based on the type of uniform worn by schoolchildren.

**Materials and methods:**

The search strategy was performed in eight databases—PubMed, Web of Science, SPORTDiscus, ScienceDirect, Embase, CINAHL, Scopus, and LILACS—following PRISMA guidelines. They were selected on the basis of the following criteria: they were children and adolescents in school and used traditional schools or sports uniforms with their levels of physical activity and physical condition. Additionally, a manual search was performed on Google Scholar to include gray literature.

**Results:**

From 1,703 initially identified studies, five studies met inclusion criteria. Sports uniforms were generally associated with higher physical activity levels compared to traditional uniforms, with girls showing 14.5 additional minutes of playtime and significantly greater activity during recess and lunch periods (*p* < 0.05). Two studies demonstrated improved cardiorespiratory and muscular fitness indicators in adolescents wearing sports uniforms. Population-level data from 135 countries confirmed these individual-level findings, showing reduced physical activity compliance in countries with mandatory traditional uniform policies.

**Conclusions:**

The available scientific evidence suggests that sports uniforms facilitate higher levels of physical activity, increased playtime, and improved physical fitness indicators among school-aged children and adolescents, with particularly pronounced benefits for girls. Schools should consider adopting more flexible uniform policies that prioritize comfort and movement to support students' overall health outcomes.

**Systematic Review Registration:**

PROSPERO CRD42024571665.

## 1 Introduction

Physical inactivity is a global public health concern, contributing to 6% of deaths worldwide (13) and increasing the risk of noncommunicable diseases, including cardiovascular diseases, diabetes, cancer, obesity, and mental illnesses (14). This issue is particularly critical in children and adolescents, as childhood obesity is prevalent worldwide (15–18) and accounts for 8% of the healthcare budget in Organization for Economic Co-operation and Development countries. Promoting physical activity among children and adolescents is a key preventive factor (19), reducing the risk of cardiovascular diseases, type 2 diabetes, and obesity (14). A systematic review with meta-analysis highlighted the mental health benefits of physical activity, emphasizing its role in reducing sedentary behavior and supporting the mental health well-being of children and adolescents (20). International guidelines recommend at least 60 min of moderate-to-vigorous physical activity three times a week for children aged 5–17 years (14, 21), emphasizing that any level of activity is better than none (14). However, despite these guidelines and the implementation of numerous physical activity programs, physical inactivity levels among children and adolescents remain alarmingly high worldwide (22).

Understanding the relationship between physical activity, fitness, cognitive function, and academic performance in children is of growing interest (1). Some studies have suggested that school-associated physical activity may benefit health and promote academic performance (2). Multiple factors influence physical activity levels in children and adolescents, including socioeconomic conditions (3–6). Research has shown that low socioeconomic status is associated with reduced physical activity levels (7–9), contributing to increased morbidity and mortality rates (10). The effects of socioeconomic disparities are long-lasting, with physical inactivity often beginning in childhood and adolescence and persisting into adulthood (11, 12). Identifying barriers to physical activity is therefore essential, as perceived barriers increase the likelihood of physical inactivity (23).

Schools can either facilitate or act as barriers to physical activity, particularly when academic activities create tension with opportunities for movement (24, 25). Common school-related barriers include bullying during recess, a lack of play facilities, the absence of policies promoting physical activity, limited space, inadequate clothing, and insufficient teacher support (26, 27). One specific and modifiable barrier is the school uniform (28), which is mandatory in many institutions worldwide. While uniforms have documented advantages, such as improving attendance (29), fostering school spirit, enhancing belonging, promoting discipline (30), and supporting health and safety (31), they also have certain drawbacks. Traditional uniforms are typically more formal and restrictive in nature, which may limit physical activity during school hours. In contrast, sports uniforms consist of athletic wear that allows greater freedom of movement. It has also been reported that mandatory uniforms could negatively impact low-income students, contributing to dropout rates (32).

Several studies have compared traditional uniforms with sports uniforms to assess their impact on physical activity levels (33). Cristi-Montero et al. (34) reported that traditional uniforms offer no advantages for promoting physical activity and recommend that sports uniforms be adopted to increase physical activity during school days. The evidence suggests that traditional uniforms reduce physical activity levels, particularly among girls (35, 36). Studies indicate that implementing sports uniforms is a low-cost, easily implementable intervention with the potential to increase physical activity levels, especially among girls (25, 37).

To our knowledge, no previous review has analyzed differences in physical activity and physical fitness related to the uniform type. This systematic review aims to identify association in physical activity levels and physical fitness based on the type of uniform worn by schoolchildren.

## 2 Material and methods

A systematic review involving the collection and analysis of analytical data from several electronic databases was performed in accordance with the Preferred Reporting Items for Systematic Reviews and Meta-Analyses (PRISMA) checklist (38).

The protocol is registered in the Prospective International Registry of Systematic Reviews (PROSPERO) with the following registration number: CRD42024571665.

### 2.2 Criteria for considering studies for this review

#### 2.2.1 Types of studies

We included observational (cross-sectional and longitudinal) and experimental studies published in Spanish, English, and Portuguese. Furthermore, we included studies that compared the use of a traditional school uniform and a sports uniform.

#### 2.2.2 Types of participants

We included school-age children and adolescents. We excluded participants with metabolic, skeletal muscle, or mental health-related pathologies.

### 2.3 Types of outcomes

#### 2.3.1 Primary outcome

Studies eligible for inclusion in this review were required to assess physical activity within the study population. Physical activity can be measured in several ways, such as the proportion of individuals who are active or inactive, the frequency of physical activity, the percentage meeting activity guidelines, or the percentage engaged in active travel. Both objective methods (e.g., accelerometers, pedometers) and subjective methods (e.g., self-report questionnaires, diaries) were considered for measurement (39).

#### 2.3.2 Secondary outcomes

Our secondary outcomes were as follows:

- Cardiorespiratory fitness.
- Muscular fitness.

#### 2.3.3 Search methods for the identification of studies

We attempted to identify all potential studies regardless of publication status. We searched the following databases for relevant studies: PubMed, Web of Science, SPORTDiscus, ScienceDirect, Embase, CINAHL, Scopus and LILACS.

The search strategy used included the following terms: (school uniforms OR traditional uniforms) AND (physical activity OR physical education OR exercise) AND (children OR adolescents OR schoolchildren).

We checked the references of relevant studies to identify additional studies and Google Scholar.

### 2.4 Data collection and analysis

#### 2.4.1 Selection of studies

For data management, we use Rayyan software. Two review authors (AP-L and RY-S) independently screened the title, abstract and keywords of each record identified in the electronic database searches. We retrieved the full-text of all potentially relevant studies. The two review authors (AP-L and RY-S) independently applied the inclusion criteria to each of these studies to determine their eligibility for inclusion. We resolved any disagreements through discussion or by consulting the third review author (JO-A) where appropriate. We attempted to contact the authors of the included studies to obtain key missing data as needed.

#### 2.4.2 Data extraction

Two review authors (AP-L and RY-S) independently extracted data from studies identified in the search and screening process as eligible for inclusion in the review. We entered the data into a data extraction form that included information about the study year of publication, study population, country, school uniform, intervention or exposure and outcome measures. Two authors compared the extracted data to identify errors, resolving any conflicts through discussion or, when necessary, by consulting the third review author (JO-A).

### 2.5 Assessment of risk of bias in included studies

The risk of bias for the included studies was evaluated independently by two authors (JG and RY-S). The Joanna Briggs Institute's tools (JBI) for analytical cross-sectional studies was used for cross-sectional studies to assess the methodological quality of the studies and to determine the extent to which a study has addressed the possibility of bias in its design, conduct and analysis (40). Possible disagreements were discussed between the same authors. The third author (JO-A) was consulted if no agreement was reached. The risk of bias of the study was judged as follows: high risk if at least two domains were considered high risk (no); moderate risk if one domain was considered high risk or if two or more domains were considered unclear; and low risk if no domains were considered high risk (yes). For cluster-randomized trials, the authors independently assessed the risk of bias via text from study reports to judge the risk as high, low, or unclear for six study features on the basis of the Cochrane Risk of Bias 2.0 tool for cluster-randomized trials (41). These features include the randomization process, timing of participant identification or recruitment, deviations from intended interventions, missing outcome data, measurement of outcomes, and selection of reported results. For trials with a crossover design, we used the Cochrane Risk of Bias 2.0 tool for crossover trials (41). This tool assesses the same features as the tool for cluster-randomized trials, except for the timing of participant identification or recruitment. Instead, it evaluates the period and carryover effects of the intervention.

## 3 Results

### 3.1 Description of studies

For details of the characteristics of the included studies, see Table 1.

**Table 1:** Search strategy.

Database	Search strategy	Limits
Pubmed, Web of Science, Scopus, LILACS, Embase, SPORTDiscus, CINAHL, Embase, ScienceDirect	School uniforms OR tradicional uniforms AND physical activity OR physical education OR exercise AND children OR adolescents OR schoolchildren	Publication date from inception to 01/06/2024

Searching other resources.

#### 3.1.1 Results of the search

**Figure 1:**
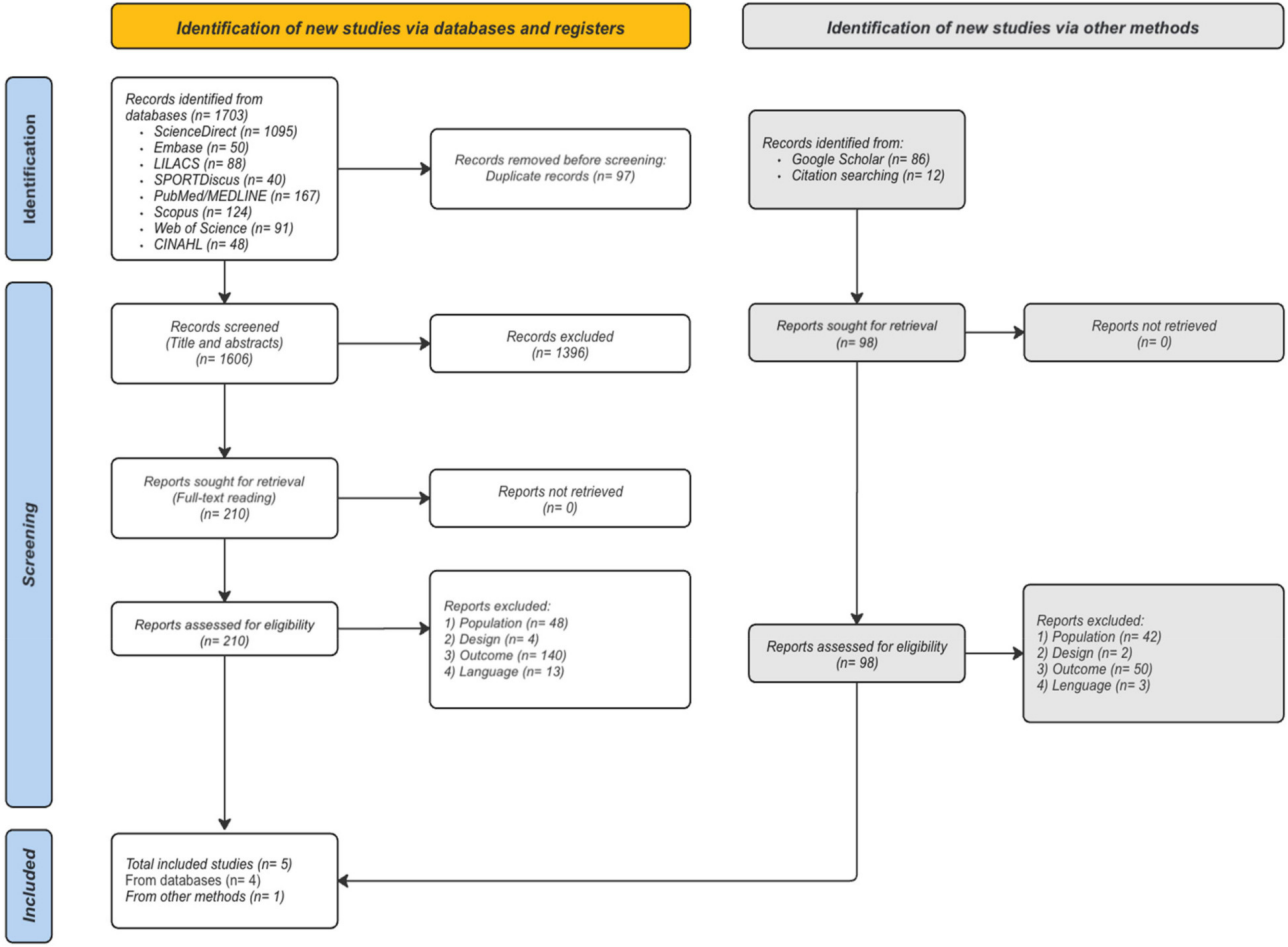
Flow diagram of the literature search and selection criteria adapted from PRISMA (38).

The literature search conducted up to June 2024 across databases resulted in 1,703 records. After removing duplicates, 1,606 records remained. Title and abstract screening identified 210 records for full-text review. After all 210 records were assessed, we identified five studies that met the inclusion criteria and were included in the review (25, 34, 35, 42, 43). Additionally, we found 96 records through Google Scholar and citation searching, but none were included. The results of the search are presented in a PRISMA flow diagram (Figure 1).

#### 3.1.2 Included studies

Five studies including 1,098,972 participants were eligible for inclusion (25, 34, 35, 42, 43). One of the studies used a crossover design (35), where participants wore their standard winter school uniform for two weeks, and after this period, participants wore their sports uniform for two more weeks. Another study (25) used a cluster randomized controlled trial, where participants wore a sports uniform or their usual traditional uniform. The other two studies (34, 42) used a cross-sectional design and compared participants wearing traditional uniforms with those wearing sports uniforms across several outcomes. Finally, a cross-sectional study investigated whether school uniforms are associated with population-level gender inequalities in physical activity levels (43).

#### 3.1.3 Settings and participants

The studies were conducted in school settings in Australia (35), England (25), Chile (34, 42), and multiple other countries (43). Only one study focused exclusively on girls (25). Children and adolescents were included in the studies.

#### 3.1.4 Interventions and exposures

All studies compared the use of a traditional uniform with a sport uniform. Details of each uniform and the procedures can be found in Table 2.

**Table 2:** Characteristics of the included studies.

Study	Participant characteristics			Design/objectives	School uniform characteristics/Intervention or exposure	Outcomes^a^	Results
	Country	Population	Age				
Norrish et al. (35)	Australia	60 children (60% males)	10.48 ± 0.53	Crossover trial/Examine the effect of school uniforms on the amount, and perceived intensity of physical activity, performed by 10-year-olds during play breaks in school.	The study does not provide details of the uniforms used, only describes that a winter uniform and a sports uniform were used/In Weeks 1 and 3 participants wore their standard winter school uniform and in Weeks 2 and 4, participants wore their sports uniform.	Physical activity (number of steps taken during recess and lunchtime each day through Yamax Digi-Walker SW200 pedometer).	Girls, but not boys, took more steps during play breaks when wearing sports uniform compared to winter uniform. Steps with winter uniform: 933.3 ± 271.8, Steps with sport uniform: 1,134.1 ± 271.9, *p* = 0.006.
Nathan et al. (25)	England	42 schools, 1,847 (girls)	Intervention group: 8.22 ± 0.70 and control group: 8.15 ± 0.67	Cluster randomized controlled trial/assess the impact of a physical activity enabling uniform intervention (shorts, polo shirt and sports shoes) on girls' moderate-to-vigorous physical activity and total physical activity.	Traditional school uniform, which for girls consisted of a skirt or dress and for boys, shorts and a button up shirt with black leather shoes for both Uni-sex sports uniform consisting of the same short pants and polo-shirt for girls and boys that was worn with sports shoes./School staff, supported by researchers, asked students to wear their sports uniform on a nonsports day to compare physical activity levels between wearing sports uniforms and traditional uniforms. The “intervention day” was randomly selected, excluding the regular sports day.	Physical activity (using wrist-worn ActiGrap GT3X + and GT9X accelerometers during school hours. Counts per minute was calculated by dividing the total accelerometer counts by the minutes of wear time).	No significant differences between groups on the change in girls moderate to- vigorous physical activity and total physical activity from the usual uniform day to intervention day. However, exploratory analysis found small improvements in light intensity physical activity (1.47, 95% CI −0.06, 3.00, *p* = 0.059) and reductions in sedentary activity (− 2.23, 95% CI −4.49, 0.02, *p* = 0.052).
Cristi-Montero et al. (34)	Chile	988 (52.6% males)	11.8 ± 1.2	Cross-sectional/To compare academic achievement, cognitive performance, playtime, bullying, and discrimination in adolescents according to traditional uniforms and sports uniforms worn at school, while simultaneously exploring the influence of the school vulnerability index.	Traditional uniform: for male adolescents was outlined as a polo shirt or shirt (with school necktie), sweater or blazer, and trousers, and for girls, a skirt and blouse, and sweater or blazer, both with school shoes (usually black leather). Sports uniforms: consisted of adolescents (both boys and girls) wearing mainly sportswear such as polo shirts or t-shirts and sport or short trousers (jeans were included as well in this category due to their generalized wearing), and sneakers/Wear traditional uniform or sport uniform.	Playtime (Chilean physical activity questionnaire).	64.1% of participants reported that traditional uniforms affected their physical activity (traditional uniforms: + 8 min vs. sports uniforms: + 20 min).
Cristi-Montero et al. (42)	Chile	8,030 (52.6%) males)	13.8 ± 0.7	Cross-sectional/to compare cardiorespiratory and muscular fitness and waist-height-to-ratio in adolescents according to school uniforms.	The traditional uniform was defined as a polo shirt or shirt (with school tie), sweater or blazer, and pants, and for girls, a skirt and blouse, and sweater or blazer, both with school shoes (usually black leather). At the same time, sport uniform was defined as sports clothing such as polo shirts or t-shirts and sports pants or shorts (jeans were also included in this category due to their widespread use and sports shoes)/Wear traditional uniform or sport uniform.	Cardiorespiratory fitness (CRF) Muscular fitness (MF)	Overall, sports uniforms (SU) were linked to higher CRF (*p* < 0.001) than the traditional uniform (TU). Boys from private schools wearing SU presented higher CRF (*p* = 0.016; effect size = 0.37), and a positive trend was observed for MF (*p* = 0.645; effect size = 0.21). In subsidized, a trend was found in CRF (*p* = 0.005; effect size = 0.16). Girls wearing SU from private schools showed a positive trend in CRF (*p* = 0.167; ES = 0.28). Trends in WHtR were found in both sexes from private (*p* = 0.555; effect size = 0.24; *p* = 0.444; effect size = 0.25, respectively).
Ryan et al. (43)	Mutiple countries (*n* = 135)	1,089,852	n/a	Cross-sectional/assess whether school uniforms are associated with population-level gender inequalities in physical activity, and whether associations differ by school level, country/region income, and assessment method.	School uniform was defined as “a consistent and standardized set of clothes that all students are required to wear during school hours”. Each country was categorized based on whether the majority (more than 50%) of primary and secondary schools require uniforms.	Physical activity was measured in each study using surveys or devices.	In countries or regions where more than 50% of schools require uniforms, compliance with physical activity guidelines was lower across all genders (median: 16.0%, interquartile range: 13.2%–19.9%, *N* = 103) compared to countries or regions without uniforms (median: 19.5%, interquartile range: 16.4%–23.5%, *N* = 32) (z = 3.04, *p* = 0.002). (N represents the number of countries, regions, and studies included; *n* represents the sample size or number of participants).

a Outcomes relevant to our review aim.

#### 3.1.5 Outcomes

##### 3.1.5.1 Physical activity

Two trials and one cross-sectional study reported results related to physical activity levels. One of them counts the number of steps taken during recess and lunchtime each day through the Yamax Digi-Walker SW200 pedometer (35) and finds that girls, but not boys, took 2008.8 more steps during play breaks when wearing a sports uniform than when wearing a winter uniform. Another study (25) used wrist-worn ActiGraph GT3X+ and GT9X accelerometers during school hours. Counts per minute were calculated by dividing the total accelerometer count by the minutes of wear time. No significant differences were found between the groups. Cristi-Montero et al. (34) reported that more than half of adolescents declared that traditional uniforms negatively affect their physical activity.

##### 3.1.5.2 Cardiorespiratory fitness

The study by Cristi-Montero et al. (34) evaluated this outcome with the 20-m shuttle run test and reported a cross-sectional association between the use of sports uniforms and higher cardiorespiratory fitness (*p* < 0.001) compared with the use of traditional uniforms.

##### 3.1.5.3 Muscular fitness

Muscular fitness was reported by Cristi-Montero et al. (42). It was evaluated with the standing long jump test, and no cross-sectional associations were found between the use of a sports uniform or traditional uniform in muscular fitness.

#### 3.1.6 Risk of bias in the included studies

**Figure 2:**
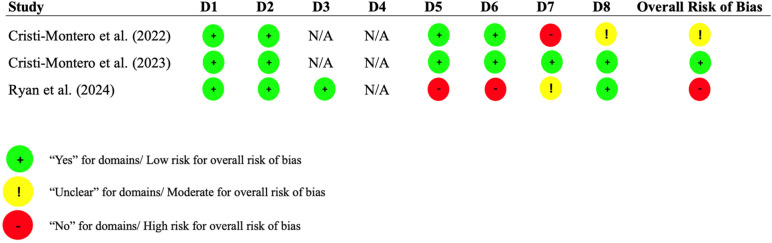
The joanna briggs institute (JBI) critical appraisal checklist for analytical cross-sectional studies. **D1:** Were the criteria for inclusion in the sample clearly defined?; **D2:** Were the study subjects and the setting described in detail?; **D3:** Was the exposure measured in a valid and reliable way?; **D4:** Were objective, standard criteria used for measurement of the condition?; **D5:** Were confounding factors identified?; **D6:** Were strategies to address confounding factors stated?; **D7:** Were the outcomes measured in a valid and reliable way?; **D8:** Was appropriate statistical analysis used?; **N/A**, not applicable.

**Figure 3:**
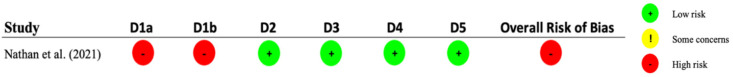
The cochrane risk of bias 2.0 tool for cluster-randomized trials. **D1a:** randomization process; **D1b:** timing of identification or recruitment of participants; **D2:** deviations from intended interventions; **D3:** missing outcome data; **D4:** measurement of the outcome; **D5:** selection of the reported result.

**Figure 4:**
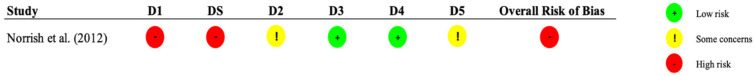
The cochrane risk of bias 2.0 tool for crossover trials. **D1:** Randomization process; **DS:** period and carryover effects; **D2:** deviations from intended interventions; **D3:** missing outcome data; **D4:** measurement of the outcome; **D5:** selection of the reported result.

Figure 2 presents the results of the risk of bias assessment using the JBI Critical Appraisal Checklist for analytical cross-sectional studies. Figure 3 shows the findings from the Cochrane Risk of Bias 2 tool for cluster-randomized trials, whereas Figure 4 displays the results from the Cochrane Risk of Bias 2 tool for crossover trials. Among the cross-sectional studies, one was classified as high risk (43) because of the lack of consideration for confounding factors in the analysis. In the trial group, neither of the two studies reported whether allocation concealment was maintained, leading to both being judged as having a high risk of bias.

## 4 Discussion

**Figure 5:**
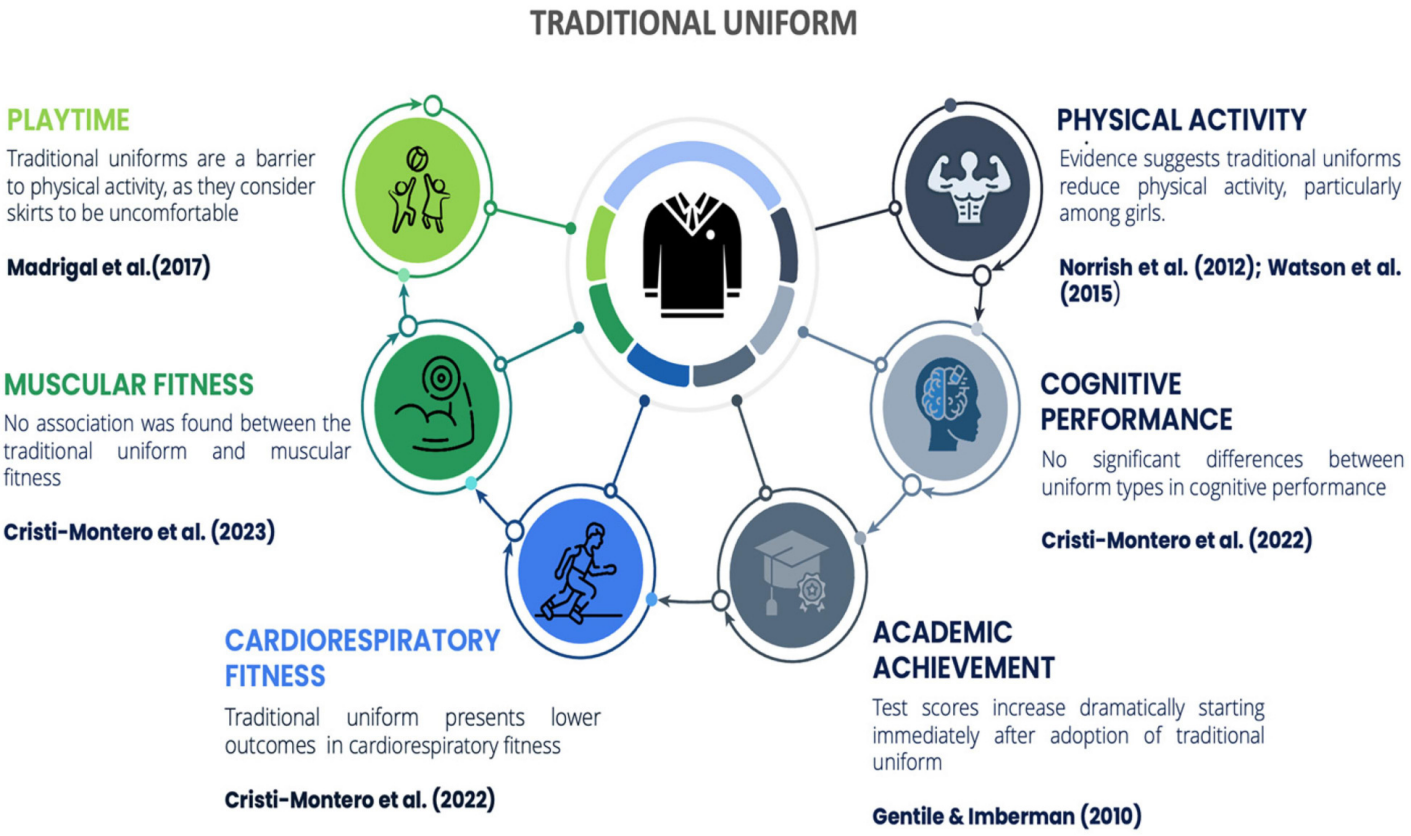
Main outcomes associated with the use of traditional school uniforms.

**Figure 6:**
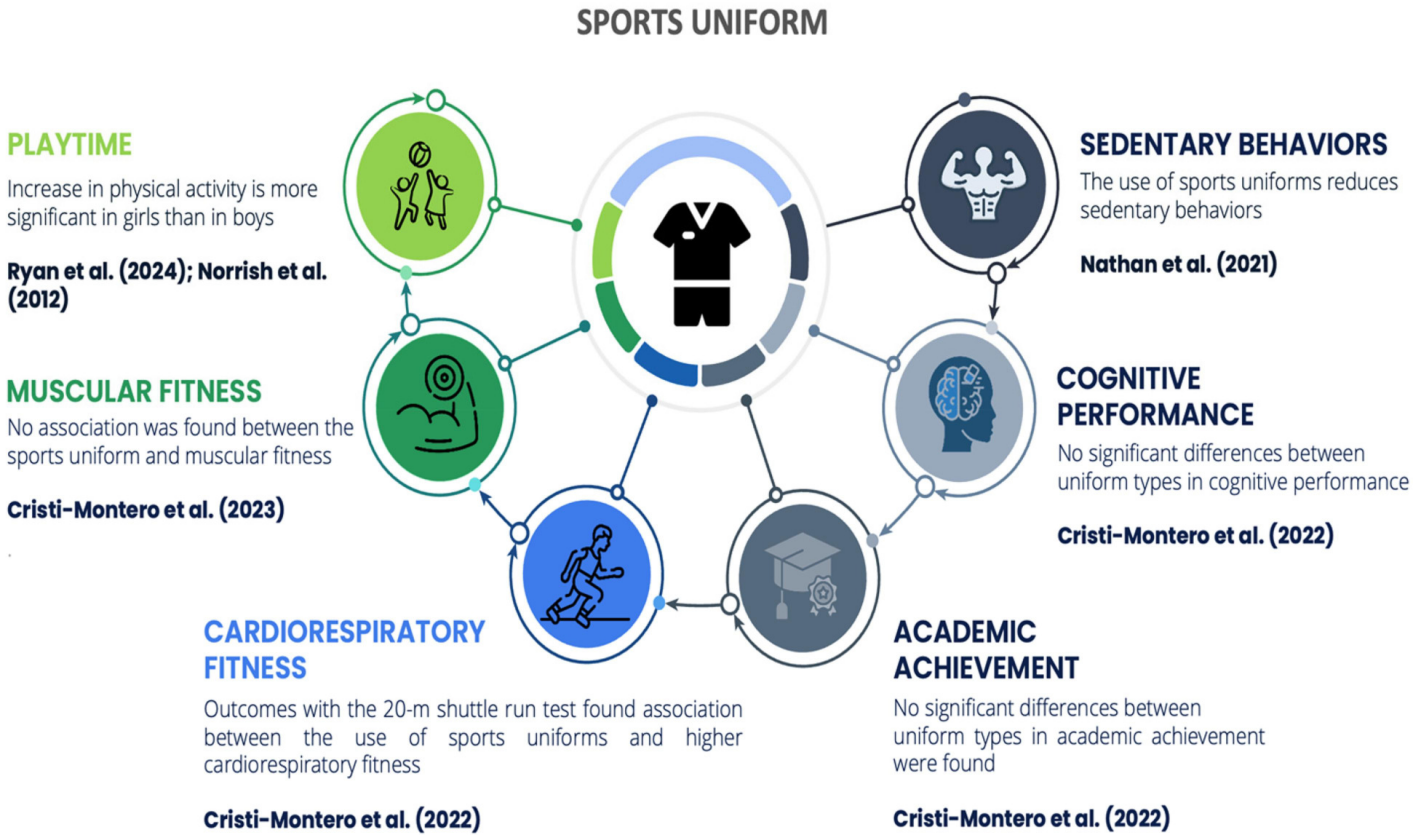
Main outcomes associated with the use of sports uniform.

The objective of this systematic review was to identify differences in physical activity and physical fitness based on the type of uniform commonly worn by schoolchildren. The results show a trend toward the benefits of sports uniforms over traditional uniforms in the variables investigated (Figures 5, 6).

Wearing sports uniforms instead of traditional uniforms could have several benefits for children and adolescents (34, 37). Firstly, sports uniforms are designed to be more comfortable and flexible, which encourages greater physical activity and participation in sports (26, 37). This increased activity can lead to improved aerobic capacity and muscular strength (42). Secondly, sports uniforms reduce movement restrictions, allowing children to engage in more vigorous exercise without feeling constrained by their clothing (25, 26). Lastly, wearing sports uniforms can foster a positive attitude towards physical fitness, making children more likely to enjoy and participate in physical activities regularly (37).

In relation to sedentary time, a study conducted by Nathan et al. (25) revealed a significant reduction in sedentary behaviors among students who wore sports uniforms. Other studies report an increase in physical activity with the use of sportswear (34). Similarly, research conducted by Stanley et al. (44) indicates that sports uniforms are explicitly compatible with children's body mobility, thus motivating and helping students engage in physical activity in a comfortable, safe, and effective manner. The increase in physical activity is more significant in girls than in boys (35, 43).

From the perspective of reduced movement, there is evidence that traditional uniforms restrict movement, leading to a reduction in the amount of physical activity that children engage in (36, 43). There is some contradictory evidence. The study conducted by Stanley et al. (45) associates variables that affect the amount of physical activity during recess and lunch, establishing a positive correlation between traditional uniform use and physical activity, an effect observed in girls but not in boys. Nevertheless, among their final recommendations, they suggest changing traditional uniforms as a strategy to increase physical activity to facilitate movement. Another aspect studied is the material and design of the uniforms. While sports uniforms have advantages over traditional uniforms, they can sometimes also be a barrier to physical activity (46), especially for girls, who prefer the use of their “sporty” uniform (37) in response to the comfort of movement that it entails (47).

With respect to cognitive parameters, only one study reported cognitive effects. The results indicate that there are no significant differences in academic or cognitive outcomes when comparing the use of traditional uniforms and sports uniforms. Therefore, they suggest the use of sports uniforms, as they offer advantages in terms of the amount of physical activity students engage in (34). The study by Gentile & Imberman (48) presents contradictory results, finding improvements in language and mathematics two years after the implementation of uniforms.

Similarly, the study developed by Baumann et al. (30) concludes that the use of uniforms in the school environment favors an improvement in discipline in daily activities and, in turn, is associated with better performance as a result of better listening. Another study conducted with Korean students revealed that wearing uniform restraints and stifles creativity, although academic performance is not affected (49). These results are consistent with another study that reported that the introduction of the uniform improved attendance and performance in students (50). Finally, a study that synthesized 800 meta-analyses of the effects related to educational outcomes concluded that the relationship between uniformity and academic achievement is insignificant (51). These diverse results related to the impact of uniforms on different domains of education validate the importance of further research.

Finally, several studies report greater benefits for girls (43, 52). A study conducted by Nathan et al. (25) revealed significant differences in physical activity during recess among girls who are part of the group wearing sports uniforms. Girls also take more steps during recess when wearing sports uniforms (35) and report that traditional uniforms are a barrier to physical activity, as they consider skirts to be uncomfortable (28).

### 4.1 Limitations

The main limitation is associated with the low number of papers found that met the criteria. Likewise, the low methodological quality of some studies and the heterogeneity of the samples evaluated do not allow us to draw solid conclusions on how uniformity is related to physical activity and fitness. Nevertheless, this is the first systematic review that evaluates the relationship between the type of school uniform and physical activity and fitness among children and adolescents.

### 4.2 Future perspectives and recommendations

Future research should aim to provide a clearer and more standardized description of how the implementation of uniforms, including their type, material, duration, color, etc., influences academic performance, physical and mental health, and other parameters of physical fitness. This would imply the development of consistent methodologies to measure the influence of uniform use in response to different contexts, countries, latitudes, genders, and ages. Similarly, it would be important to develop comparative, multivariate and longitudinal studies between students who use uniforms and those who do not, with the goal of deepening the influence on various variables related to the social context experienced by students.

## 5 Conclusions

This systematic review is the first to comprehensively demonstrate that sports uniforms are associated with increased physical activity levels and physical fitness compared to traditional uniforms in school-aged children, with particularly significant benefits for girls. Traditional uniforms represent a modifiable barrier to both physical activity and physical fitness in educational settings. Transitioning to sports uniforms offers a pragmatic, low-cost intervention that requires no additional infrastructure or training. Schools and policymakers should consider adopting sports uniforms as standard daily attire to facilitate physical activity and enhance physical fitness while addressing gender disparities in both outcomes. This simple policy change could meaningfully contribute to meeting international physical activity guidelines and improving public health outcomes in children and adolescents. Future research should examine long-term effects on both activity and fitness, underlying mechanisms, and cost-effectiveness of this intervention across diverse settings.

## Data Availability

The original contributions presented in the study are included in the article/Supplementary Material, further inquiries can be directed to the corresponding author.
